# Community-based palliative care service provision framework (PALLIUM framework) in a resource-limited setting

**DOI:** 10.3332/ecancer.2026.2120

**Published:** 2026-05-07

**Authors:** Deepak Sudhakaran, Anoushka Arora, Smriti Rana, Amirtha Thampi, Sunilkumar, Parth Sharma

**Affiliations:** 1 Trivandrum Institute of Palliative Sciences, Pallium India, Trivandrum, Kerala 695009, India; 2 Association for Socially Applicable Research (ASAR), Pune, Maharashtra 411007, India

**Keywords:** palliative care, health system delivery, community health care

## Abstract

**Background:**

Palliative care addresses the complex needs of individuals with life-limiting illnesses (LLIs), encompassing physical, psychological, social and spiritual dimensions of suffering. In India, access to such care remains extremely limited, with less than 4% of those in need receiving it. Existing services often focus narrowly on pain relief, neglecting the broader concept of ‘total pain’. Moreover, community-based delivery models that address these holistic needs within resource-constrained environments are largely missing.

**Case presentation:**

This case report describes the development and structure of a community-based palliative care framework implemented by Pallium India, a leading non-profit organisation in the field. Using a modified Delphi method, the framework was developed through iterative discussions with clinical and non-clinical team members, incorporating expert consultations and consensus-building exercises. The resulting model outlines a decision-making flowchart for identifying eligibility, determining care pathways and integrating psychosocial support. Care is delivered through home visits, outpatient clinics, inpatient units or telehealth, depending on the severity of symptoms and geographical access. The framework includes provisions for individuals with diagnostic uncertainty, linking them to higher centers when feasible or offering provisional support. It actively addresses non-medical needs through interventions such as education support, housing repairs and vocational rehabilitation. Social officers assess and address financial and community-based needs and referrals are made to local services when appropriate.

**Conclusion:**

The Pallium India framework presents a comprehensive, equitable and context-sensitive approach to community-based palliative care. It emphasises early identification, telehealth integration, interdisciplinary care and strong community linkages. While not yet formally evaluated for outcomes such as quality of life or cost-effectiveness, the framework represents a scalable and sustainable model for resource-limited settings. Its design aligns with public health principles of equity, community participation and multisectoral collaboration, offering a promising strategy to bridge the current palliative care gap in India and other low-resource regions.

## Introduction

Palliative care is recognised as a fundamental right to health [1]. It includes the provision of physical, social, psychosocial or spiritual support to people with life-limiting illnesses (LLIs) and their families [2]. LLIs can be defined as illnesses that are progressive and fatal, and whose progress cannot be reversed by treatment [3]. Despite being an integral part of health care delivery, access to palliative care is severely lacking. As of 2020, only an estimated 14% of people worldwide who need palliative care receive it [1]. While more than 73 million people experience serious health-related suffering (SHS) that requires palliative care worldwide, only 12% of all people and 2% of children with SHS receive it [4]. In India, a country with 1.46 billion people, less than 4% of those in need can access these services [5]. The unmet need for palliative care in Indian patients with end-stage cancers only was reported to be as high as 98.3% [6].

While palliative care ideally looks beyond the medical needs of the people, the discussion around it is often restricted to access to medical care, especially pain relief through morphine and end-of-life care. While this alleviates the physical pain, it overlooks the ‘total pain’ experienced by the person. ‘Total pain’ can be defined as the suffering that encompasses all of a person’s physical, psychological, social, spiritual and practical struggles [7]. Adequate pain relief cannot be obtained until all components of total pain are addressed [8]. Total pain can be addressed by patient- and family-centered care, interdisciplinary team approach, comprehensive assessment, continuity of care and frequent reassessment and adaptation of care plans [9–11]. Launched in 2012, the National Programme for Palliative Care in India promotes community-based delivery of services but primarily emphasises the medical aspects of care, with limited focus on addressing the psychosocial needs of individuals with LLIs [12].

Presently, many countries in the Global North, including the United States, Australia and Canada, have frameworks for delivering palliative care at the community level [13–15]. In contrast, most of India’s palliative care is delivered by Non-Governmental Organisations (NGOs). They help bridge the gap in areas where healthcare infrastructure is particularly limited. However, there is a lack of a community-level framework of palliative care delivery that addresses not just physical pain but total pain. As palliative care in India is primarily delivered by NGOs, the resource-limited setting necessitates a framework that balances available resources with the needs of patients in the community.

This case report details a community-based palliative care service delivery framework that was developed and is used by Pallium India, a leading not-for-profit palliative care organisation in India.

## Background of the organisation

Pallium India is a nationally registered charitable trust formed in 2003 and is based in Trivandrum, Kerala [16]. Pallium India created the Trivandrum Institute of Palliative Sciences as its training, research and clinical demonstration unit in 2006. In 2012, it was designated a World Health Organisation (WHO) collaborating center for Training and Policy on Access to Pain Relief for the South-East Asia Region, and by 2016, the organisation had supported the initiation of palliative care services in 21 Indian states and union territories.

Pallium India’s vision is to stop needless health-related suffering to enable people to wake up to life, love and hope. It aims to achieve this through integrating quality palliative care into healthcare by collaborating with and empowering care seekers, care providers and communities. Pallium India works on the three-pronged model of ‘Demonstrate, Educate and Facilitate’. The ‘Demonstrate’ component includes the direct provision of care through home-based services, rehabilitation, link centers, inpatient and outpatient care. ‘Educate’ focuses on building capacity through virtual and on-site certificate and fellowship programs. The ‘Facilitate’ component involves working with government and public stakeholders to influence policy, integrate palliative care into education, improve access to pain relief through better legislation and promote initiatives like pain-free hospitals, while also engaging with international agencies, working on guideline development groups and advocating for a decolonised framework for palliative care.

The services offered by the organisation are free of cost and are funded by various corporate social responsibility (CSR) initiatives, high-net-worth individuals, microdonations and periodic fundraising events. In 2023, Pallium provided support to 5,278 patients, with 37,260 patient contacts and over 10,000 home visits. Their telehealth services connected with 2,253 individuals and supported 954 people. Additionally, they provided educational support to 385 children and monthly food kits to 185 families. Their education wing trained 714 healthcare professionals and 148 stakeholders virtually, and 113 professionals through classroom training. Through such trained individuals, palliative care was provided to about 118,400 patients [17].

## Development of the Pallium framework

A modified Delphi research method was employed to systematically develop the community-based palliative care service delivery framework used by Pallium India. The process involved a combination of structured group discussions, consensus-building sessions and expert consultations to ensure a participatory and balanced approach.

The method included four formal, face-to-face group discussions, each guided by a trained moderator with expertise in palliative care systems development and group facilitation. Both open-ended and closed-ended questions were used to elicit detailed responses and identify points of agreement and divergence.

The first round of discussions was conducted on 20th October 2022 with a panel of eight senior team members, including the Chief Executive Officer (CEO), Medical Director, Executive Director, Additional Medical Director and team leads from the departments of Clinical Services, Social Work, Community Engagement and Palliative Care Facilitation. This session focused on identifying the core components of palliative care delivery relevant to the Indian community setting.

After the first meeting, several key points emerged for Pallium’s delivery model. First, patient registration criteria were established to focus primarily on individuals with diagnosed LLIs or those who were bedridden, with priority given to those experiencing ongoing physical SHS. However, a critical distinction regarding an acute life-threatening condition needed to be made. While these technically fall under SHS, the consensus recognised that accepting such patients into a standalone palliative care unit without access to emergency interventions could inadvertently deny life-saving measures. For instance, a patient presenting with severe chest pain might receive symptomatic relief through morphine administration, but would have been treated appropriately in an emergency department setting.

Second, all patients must have an established diagnosis at the time of registration, with necessary investigations completed prior to referral to palliative care services. Pallium typically refers patients lacking diagnostic clarity to the nearest appropriate and affordable health care facility. However, when patients and caregivers explicitly decline further diagnostic workup and request palliative care exclusively, services may be provided following informed consent documentation.

Third, lifestyle-related conditions and general geriatric care needs were excluded from the Palliative Home Care and Telemedicine programs, with consideration given to developing a separate Primary Healthcare initiative staffed by personnel trained in geriatric care principles. Finally, to minimise repeated efforts and optimise resource utilisation, Pallium maintains a dynamic directory of affiliated community-based palliative care providers, enabling appropriate referral to active local facilities when available. For issues predominantly social in nature, coordination with local organisations or individuals engaged in social welfare was deemed appropriate.

The second round took place on 28th October 2022 at Pallium India and expanded the panel to include 30 staff members. This larger group represented a diverse mix of personnel across clinical and non-clinical services, including those involved in direct care, support functions and administrative roles. The collective aimed to answer two key questions:

1. *What does palliative care mean in our context?*
2. *How do we, at Pallium India, envision delivering it to our communities?*

On 21st November 2022, the outcomes from the first two discussions were shared and reviewed for consensus within both original groups. This iterative feedback loop ensured cross-validation of ideas and encouraged transparency and inclusivity in decision-making. Importantly, two distinct groups were intentionally maintained throughout the process to enhance participation, minimise hierarchical influence and avoid information or response bias.

To further refine the emerging framework, individual expert consultations were also conducted between the moderator, CEO, Medical Director and Executive Director. These sessions enabled deeper discussion on areas of divergence and helped incorporate strategic and operational insights into the final design of the framework.

## Description of the Pallium framework

**Figure 1.**
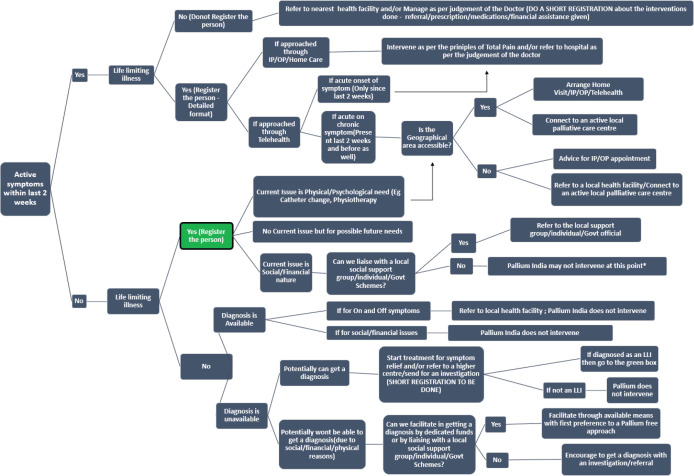
Community-Based Palliative Care Service Provision Framework - PALLIUM framework (*Patient will not be receiving any support from Pallium India till the time a dedicated fund becomes available for such social/financial support AND/OR till they develop active symptoms arising out of the LLI they suffer from.)

The modified Delphi process led to the development of a decision-making flowchart to determine eligibility and pathways for community-based palliative care service delivery. The framework is represented in Figure 1.

### Entry point: identifying active symptoms and LLIs

The framework begins by assessing whether the person has had any active symptoms in the past 2 weeks. If the answer is yes, the next step is to determine whether the individual has an LLI. After deliberate discussion, Pallium designed its operational definition of LLIs. An LLI is any illness that limits either the quantity (span) or quality of life or both. It will have either one or more of the following characteristics: conditions for which treatment may be feasible but can fail, conditions where premature death is inevitable, but where there may be long periods of participation in normal activities, progressive conditions without curative treatment options, where treatment is exclusively palliative and commonly extends over many years, and irreversible but non-progressive conditions which may or may not cause premature death [18]. The LLIs for which palliative care services were offered consisted of both cancer and non-cancer illnesses [19]. If both conditions are met, the person is eligible to receive palliative care services from the organisation and is registered for the same. However, if the person does not have an LLI, they are not registered and are instead referred to the nearest health facility for appropriate care. In such cases, only a short registration may be done to document referrals, prescriptions and interventions. If the person does not have active symptoms but has a confirmed LLI, they are still eligible and are registered for palliative care services, which may be needed in the future. If neither condition is met, the individual is not registered and is referred to regular healthcare services.

### Pathways based on mode of contact

Once a person with active symptoms and LLI is registered, the person and their family become beneficiaries of the services being offered by the organisation. The mode of care is determined by the severity of the patient’s symptoms and the point of contact. Care may be delivered at the patient’s home within the community, through regular outpatient clinics or via admission to an inpatient unit when necessary. If they approach via telehealth, further assessment is done to check whether the symptoms are new (acute) or an acute worsening of chronic symptoms. For individuals with acute symptoms, the framework first assesses the accessibility of the geographical area. If the area is accessible, care is arranged through home visits, outpatient appointments or inpatient admissions as appropriate. If the area is not accessible, the individual is referred to a nearby healthcare facility or an active local palliative care center, along with guidance to seek in-person consultations when feasible. Based on the complexity of the condition and the need for specialist intervention, referrals may also be made to government or private super-specialty hospitals.

### Service delivery

When symptoms are chronic, the current issues are classified into two categories. The first includes physical or psychological needs such as symptom management, catheter changes, physiotherapy or counseling, which are addressed directly by the appropriate team member, such as a doctor, nurse or clinical psychologist. The second includes social or financial concerns, in which case a social officer assesses the situation and explores options such as involving volunteers, connecting with local support groups or facilitating access to relevant government schemes when available and applicable.

Regardless of the mode of service delivery, Pallium India follows the principles of assessing ‘Total Pain’, viewing the patient and family as a single unit of care. Its interventions go far beyond medical treatment, addressing the wide spectrum of physical, emotional, social and spiritual suffering experienced by individuals with LLIs. The organisation actively works to support children in such families by facilitating continued access to education, recognising that a child’s disrupted schooling can have long-term consequences on their well-being. In paediatric palliative care settings, deliberate attention is paid to the healthy sibling of a sick child, as they are often unintentionally sidelined, resulting in emotional and behavioural issues. In cases where patients live in unsafe or dilapidated housing that compromises their quality of life, Pallium India arranges for repairs or improvements to ensure a more dignified and comfortable living environment. Additionally, vocational rehabilitation is provided to empower patients or their family members with skills and opportunities to earn a livelihood, helping them maintain financial independence and dignity even in the face of serious illness. This holistic, person-centered approach ensures that the care provided is not only comprehensive but also compassionate and responsive to the real-life needs of those affected.

If there are available social or governmental supports, the person is referred accordingly. If no local liaison is feasible or if a dedicated fund is not available for such social/financial support, then Pallium India may not intervene at that stage.

### Diagnostic uncertainty

For individuals suspected of having an LLI but without a confirmed diagnosis, the framework first assesses whether any past diagnosis is available. Those with a previous diagnosis are registered and managed based on their current needs as they arise. For individuals without a confirmed diagnosis, the team evaluates the likelihood of establishing one. If a diagnosis appears feasible, a provisional (short-term) registration is done, symptom management is initiated and the individual is referred to a higher center for the necessary investigations. In cases where obtaining a diagnosis is unlikely due to social, financial or physical barriers, the organisation actively works to bridge these gaps, facilitating diagnostic access through collaboration with local support groups, volunteers, government healthcare services or by mobilising funds when required. It is important to note that the unavailability of diagnostic services could arise from system-level limitations, such as service unavailability and geographical accessibility, instead of patient-level factors [20]. If, after support, the person is diagnosed with an LLI, they return to the main registration pathway for palliative care. If they are diagnoses is not a LLI, then the person is referred to appropriate healthcare services.

## Discussion

Palliative care is a key component of health care and has been gaining importance. Globally, there has been a push to develop community-based palliative care services to reach a larger population outside of hospitals [21]. This is particularly important as community-based services help address the shortcomings of inpatient consultative palliative care. This includes failing to address longitudinal needs across diverse settings of care via frequent follow-ups, which enables sustained evaluation and adjustments in their management plans. Moreover, inpatient palliative care fails to comprehensively assess the need for palliative care in patients’ natural environment, which affects patients’ symptoms, provides insufficient time to consult and fails to include palliative care early in the disease [13, 22–24]. Additionally, outpatient palliative care delivery, an integral part of community-based palliative care, helps provide greater satisfaction and comfort to care receivers and improves outcomes for caregivers, which includes a greater ability to ‘move on’ after the death of a loved one and fewer unmet needs in the five domains of caregiving explored by Given *et al* [13, 25–30].

Many high-income countries like Scotland, England, the United States, Canada, New Zealand and Australia have community-based palliative care delivery services. A common thread between these delivery models is the integration of person-centered care with a multidisciplinary approach. In contrast, low-income countries (LICs) since India is an LMIC, not LIC. lack a robust palliative care delivery model. An efficient community-based palliative care model is a necessity to overcome the challenges faced by these countries due to resource constraints. Such a model will additionally help overcome other challenges to palliative care delivery, like poor geographical access and a lack of policy and legislation [31].

Creating a sustainable care delivery model depends on certain factors like sustainable funding, policy developments, workforce training and community engagement [31]. Of these, an essential requisite of sustainability is reliable funding. Globally, palliative care financing is often characterised by a mixed-payer system [32]. On the other hand, organisations in Low-and Middle-Income Countries, such as Hospice Africa Uganda and Pallium India, have relied heavily on charitable donations and CSR funds. According to an Organisation for Economic Co-operation and Development report, ways to reliably finance the care of people with LLIs are to incentivise the promotion of palliative care and use more cost-effective interventions like home-based care [33]. The framework developed by Pallium India is not only scalable, but the multisectoral engagement also makes the framework sustainable by ensuring the availability of resources from different avenues.

Public-private partnerships (PPPs), a feature of the Pallium Framework, are another integral part of sustainability and scalability [34]. PPPs help leverage the advantages of both the private and public sectors. Private organisations have skilled management, develop better relationships between stakeholders and are innovative when it comes to health promotion [35]. Similarly, the public sector has its strengths, such as providing technical efficiency and increased scalability of services [36].

The community-based palliative care model of Kerala is economically self-sufficient through their funding mechanism, which is supported by the local self-government and the people from the community. Community-based resource mobilisation allows these centers to provide comprehensive care packages that include free medications, nursing care, medical equipment and financial support, which greatly reduces the out-of-pocket expenses for families [37]. This model demonstrates significant positive long-term effects on caregivers by reducing their multidimensional burdens [38]. Kerala’s community-based palliative care model addresses the physical, social and mental tolls experienced by families through its comprehensive home-based care approach, which eliminates the financial strain of repeated hospital visits, transportation costs and hospitalisation charges. By providing free home care by trained community volunteers and healthcare professionals, the demands of caregiving are substantially reduced. By mobilising resources and sharing caregiving responsibilities within the community, this community-driven model helps prevent the caregiving burden faced by families who manage chronic or terminal illnesses alone. Importantly, this model has benefited in improving access to palliative care in India, a resource-limited country, with near universal access in states like Chandigarh and Kerala [20]. Currently, palliative care services under the National Programme for Palliative Care are functional in 600 districts, with 2,517,663 people receiving home-based care in 2024 as per the government report [39].

The development of a framework for community-based palliative care service delivery has many policy implications. Since the Pallium framework provides a robust model for community-based palliative care, this would enable palliative care to be formally recognised as an essential part of the national health system, in alignment with the WHO’s call. Additionally, such a framework could incentivise workforce development, capacity building and can also be used to develop monitoring and evaluation frameworks, which will ensure quality assurance with regard to palliative care service delivery.

### Strengths and limitations of the framework

This framework represents a significant advancement in the delivery of community-based palliative care in India. To the best of our knowledge, it is one of the first of its kind developed within the Indian context, specifically tailored for resource-limited settings. Its design enables potential adaptation and replication in other low-resource environments globally, where gaps in palliative care access and delivery persist. One of the key strengths of the framework lies in its comprehensive, future needs-oriented approach. It not only addresses current clinical, social and logistical needs but also anticipates future care requirements, allowing for proactive registration and planning. The inclusion of pathways for individuals with diagnostic uncertainty or psychosocial barriers ensures that no one is excluded based on socioeconomic disadvantage.

Additionally, the framework emphasises community ownership and multi-level engagement. It delineates clear points where individuals, families, local communities, civil society organisations and government bodies can play an active role. This participatory approach supports decentralised, context-sensitive palliative care, which is essential in diverse Indian settings. A notable innovation is the integration of telehealth, which strengthens the reach of palliative services, especially in geographically inaccessible or underserved regions. This use of digital tools enhances continuity of care and overcomes traditional barriers to access. Importantly, the framework aligns with several core principles of public health. It promotes equity, community engagement, collaborative partnerships, social justice and sustainability, making it not just a clinical tool but a public health intervention in itself.

However, the framework also has certain limitations. First, while it was developed through a rigorous modified Delphi process involving both clinical and non-clinical stakeholders, at the time of its creation, the primary intent was not academic research but practical implementation. As a result, detailed documentation of the academic methodology, such as approval rates or consensus thresholds, is not available. Despite this, the framework followed a rigorous and iterative consultative process, as outlined in the methodology section and offers valuable insights and learning opportunities for the global palliative care community. Second, although the framework has been in use since its creation, it has not yet been formally validated through empirical research. At present, there is no data on its cost-effectiveness, impact on quality of life or long-term outcomes for patients and families. Furthermore, as with all frameworks developed through consensus, there is a risk of subjectivity and contextual bias. Future studies are needed to evaluate the implementation and effectiveness of this framework in real-world settings. Research should focus on measurable outcomes such as patient satisfaction, symptom burden, health system efficiency and community participation, to strengthen the evidence base and inform further refinement.

## Conclusion

This framework ensures a needs-based, equitable and context-sensitive approach to delivering palliative care in the community. It emphasises early registration for those with LLIs, strategic use of telehealth, incorporation of local resources and support for diagnostic access, especially for those facing social or financial challenges. It also clearly outlines when the organisation should intervene and when referral to local services is more appropriate.

## List of abbreviations

CEO, Chief Executive Officer; CSR, Corporate Social Responsibility; LICs, Low Income Countries; LLI, Life-limiting Illnesses; NGO, Nongovernmental Organisations; WHO, World Health Organisation

## Conflicts of interest

None.

## Funding

None.

## Ethics approval and consent to participate

Not applicable.

## Consent for publication

Consent for publication is not applicable for this case report as it is not a clinical case report and no patients were involved in this.

## Availability of data and materials

Not applicable.

## Author contributions

Study concept and design: Deepak Sudhakaran

Acquisition, analysis or interpretation of data: Deepak Sudhakaran, Anoushka Arora, Parth Sharma

Drafting of the manuscript: Anoushka Arora, Parth Sharma, Deepak Sudhakaran

Critical revision of the manuscript for important intellectual content: All authors

Administrative, technical or material support: Parth Sharma

Study supervision: Parth Sharma

## Reference
